# Effects of architectures and H_2_O_2_ additions on the photocatalytic performance of hierarchical Cu_2_O nanostructures

**DOI:** 10.1186/s11671-014-0726-x

**Published:** 2015-01-22

**Authors:** Xiaolong Deng, Qiang Zhang, Qinqin Zhao, Lisha Ma, Meng Ding, Xijin Xu

**Affiliations:** School of Physics and Technology, University of Jinan, 336 NanxinZhuang West Road, Jinan, 250022 Shandong Province Peoples Republic of China

**Keywords:** Cu_2_O, Hierarchical nanostructures, Photocatalytic properties, H_2_O_2_, Visible light

## Abstract

Cu_2_O hierarchical nanostructures with different morphologies were successfully synthesized by a solvothermal method using copper (II) nitrate trihydrate (Cu(NO_3_)_2_⋅3H_2_O) and ethylene glycol (EG) as initial reagents. The obtained nanostructures were characterized by X-ray diffraction (XRD), scanning electron microscopy (SEM), Brunauer-Emmett-Teller (BET) specific surface area test, and UV-vis spectroscopy. The synthesis conditions (copper source, temperature, and reaction time) dominated the compositions and the formation of crystals with different morphologies. The visible light photocatalytic properties of as-prepared Cu_2_O nanostructures were investigated with and without hydrogen peroxide (H_2_O_2_), and the effect of H_2_O_2_ were evaluated by monitoring the degradation of methyl orange (MO) with various amounts of H_2_O_2_. It was revealed that the degree of the photodegradation of MO depends on the amount of H_2_O_2_ and the morphology of Cu_2_O.

**Electronic supplementary material:**

The online version of this article (doi:10.1186/s11671-014-0726-x) contains supplementary material, which is available to authorized users.

## Background

Over the past decades, the remarkable developments of industry have induced environmental pollution which has become one of the most critical issues for the future sustainable development [1-3]. Therefore, considerable attention has been paid to fabricate photocatalytic materials with high efficiency and low cost to solve environmental problem. Metal oxide nanostructures with desired architectures have become promising candidates due to their unique properties and potential applications in many fields [4-6]. Among these metal oxides, cuprous oxide (Cu_2_O) has attracted considerable interest due to the wide applications in the fields of lithium-ion batteries [7], solar energy conversion [8,9], optical limiter [10], gas sensor [11,12], storage device [13,14], and catalysis [15,16]. Furthermore, its bandgap of 1.9 to 2.2 eV endowing them absorb visible light of solar spectrum [17], and the optical bandgaps can be fine-tuned by changing the size of nanoparticles [13], which is very helpful to degrade dye pollutants. Moreover, Cu_2_O was first explored for water splitting under visible light irradiation in 1998 [18]. Since then, many efforts have been made to investigate the factors influenced on the photocatalytic activities, and the applications have also been extended to the photodegradation of dye pollutants [3,19-23].

Since the photocatalytic activities could be strongly affected by the structural and morphological characters of materials including size, shape, and exposed crystalline plane [21,24], many approaches have been suggested to fabricate photocatalytic materials, such as chemical vapor deposition [25], hybrid laser processing and chemical dealloying [21], chemical transformation [10], electrochemical deposition [23], thermal decomposition [3], aqueous colloidal solution approach [26], and hydrothermal route [20]. Through these methods, varied shapes of Cu_2_O micro/nano-structures have been successfully synthesized, including nanowire polyhedrals [20], microcrystalline particle films [24], polyhedral microparticles [27], nanocages (nanoframes) [13,28], hollow octahedrals [29], hollow spheres [29], nanocubes [2,30], nanoplates [31], and nanoboxes [32].

In addition, hydrogen peroxide (H_2_O_2_) plays an important role in the photocatalytic activities of Cu_2_O on the degradation of dye pollutants, acting as an electron and hydroxyl radical (OH^•^) scavenger which prevents the recombination of electron-hole pairs generated during the catalysis [3,20,29]. However, there are only a few reports that investigated the effect of H_2_O_2_ amount on the degradation of dye based on Cu_2_O crystalline particle films [23,24], and almost no reports based on Cu_2_O nanoparticles. Furthermore, controversies exist about the direct photodegradation of dyes by Cu_2_O materials in the absence of H_2_O_2_ [20,27]. Therefore, we carried out the research in order to overall understand the effect of H_2_O_2_ on the photocatalytic activities of Cu_2_O particles and to clarify the controversies of Cu_2_O for direct photodegradation of dyes.

In this report, we investigated the effects of synthesis conditions on the structural and morphological features by growing Cu_2_O nanostructures through solvothermal approach. The effect of H_2_O_2_ amount on the photocatalytic activities of Cu_2_O materials was systematically studied. It was demonstrated that the compositions of the products and the formation of crystals with different morphologies could be greatly affected by the synthesis conditions. It was also revealed that the presence of different amounts of H_2_O_2_ and different Cu_2_O nanoarchitectures would play important roles in the photodegradation of methyl orange (MO).

## Methods

All the chemical reagents, purchased from Sinopharm Chemical Reagent Co., Ltd. (SCRC, China), were of analytical grade and used without further purification. The synthesis conditions used in this work are listed in Table 1. For the synthesis of Cu_2_O nanostructures, a typical procedure (S1 to S4 in Table 1) was as follows, according to the previous report [33]: Cu(NO_3_)_2_⋅3H_2_O (4 mmol, 0.9664 g) was dissolved in 80 mL ethylene glycol under vigorous stirring, then the mixture was transferred into 100 mL Teflon-lined stainless steel autoclave. After that, the autoclave was sealed and placed into oven at 140°C for several hours. Subsequently, the autoclave was cooled down to room temperature naturally. The obtained precipitants were centrifuged and washed with deionized water and ethanol several times. The final products were collected by drying the precipitants in a vacuum oven at 60°C for 12 h.

**Table 1:** **The synthesis conditions used for the preparation of Cu**_**2**_ **O nanostructures**

**Sample**	**Copper source**	**Ethylene glycol (mL)**	**Temperature (°C)**	**Time (h)**
S1	4 mmol Cu(NO_3_)_2_⋅3H_2_O	80	140	4
S2	4 mmol Cu(NO_3_)_2_⋅3H_2_O	80	140	6
S3	4 mmol Cu(NO_3_)_2_⋅3H_2_O	80	140	8
S4	4 mmol Cu(NO_3_)_2_⋅3H_2_O	80	140	10
S5	4 mmol Cu(CH_3_COO)_2_⋅H_2_O	80	140	10
S6	4 mmol Cu(CH_3_COO)_2_⋅H_2_O	80	160	12
S7	4 mmol Cu(CH_3_COO)_2_⋅H_2_O	80	180	6
S8	4 mmol Cu(NO_3_)_2_⋅3H_2_O	80	180	6
S9	4 mmol Cu(NO_3_)_2_⋅3H_2_O	80	140	3
S10	8 mmol Cu(CH_3_COO)_2_⋅H_2_O	80	160	12

X-ray powder diffraction (XRD) patterns were carried out to analyze the crystallographic structures of the products on a German X-ray diffractometer (D8-Advance, Bruker AXS, Inc., Madison, WI, USA) equipped with Cu *Kα* radiation (*λ* = 0.15406 nm). The morphologies of the products were observed by a field emission scanning electron microscopy (FESEM; FEI QUANTA FEG250, FEI, Hillsboro, USA). The Brunauer-Emmett-Teller (BET) specific surface areas of the products were investigated by N_2_ adsorption isotherm at 77 K using a full-automatic specific surface analyzer (3H-2000BET-A, Beishide Instrument, Beijing, China).

The morphology-related photocatalytic activities of as-prepared Cu_2_O nanostructures were performed with a UV-vis spectrophotometer (TU-1901, Beijing Purkinje General Instrument Co., Ltd, Beijing, China) under visible light irradiation at ambient temperature in air. The visible light was generated by a 500 W Xe lamp equipped with a cutoff filter to remove the UV part with wavelength below 420 nm. In a typical procedure, 20 mg/L MO solution was prepared by dissolving 10 mg MO in 500 mL deionized water, then 30 mg of products was added into 50 ml of as-prepared MO solution in a quartz bottle to form a suspension. Prior to illumination, the suspension was kept in dark for 30 min with stirring to reach adsorption-desorption equilibrium of MO on the surface of the Cu_2_O nanostructures. Then, different amounts of H_2_O_2_ (30 wt%) aqueous solution were added into the suspension before turning on the light. Ca. 3 mL of the dye aqueous solution was taken out at a given irradiation time interval and centrifuged to filtrate the sample powders. The concentration of the dye (MO) aqueous solution was measured by testing the absorbance properties at 464 nm in UV-vis spectra. The degradation rate of MO was defined as follows [24]:

$$ \mathrm{degradation}\kern0.24em \mathrm{rate}=\frac{C_{\mathrm{o}}-C}{C_{\mathrm{o}}}\times 100\% $$

where *C*_0_ and *C* are the absorbance value at 464 nm in UV-vis spectra before and after a given time interval of the degradation of MO, respectively.

The optical absorption behaviors of the synthesized samples were investigated by measuring UV-vis absorbance spectra directly through dissolving powders into ethanol.

## Results and discussion

**Figure 1:**
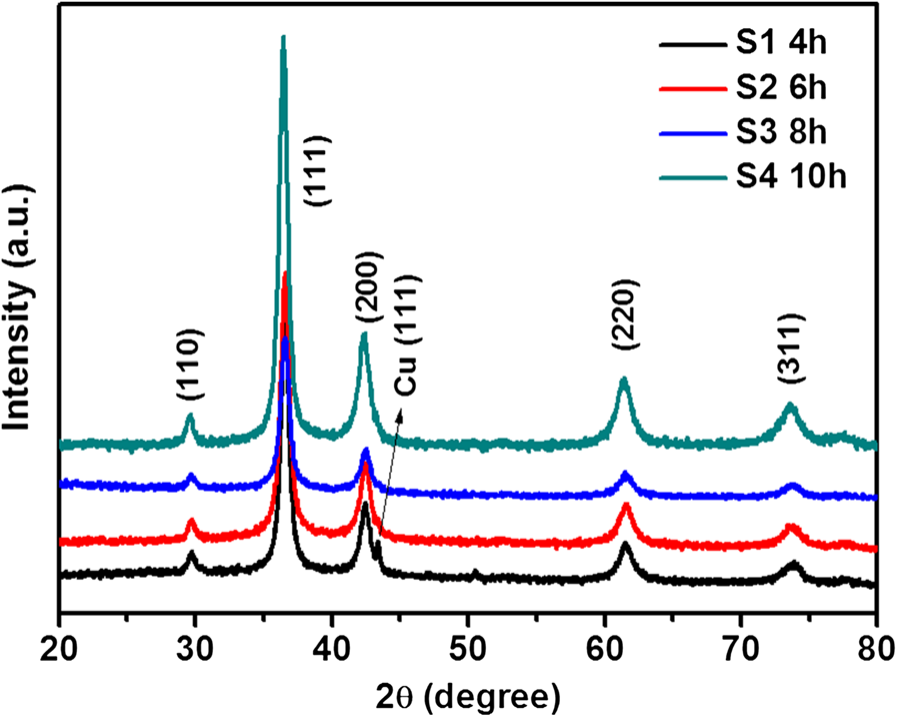
**XRD patterns of as-prepared products obtained at different reaction time.** S1 (4 h), S2 (6 h), S3 (8 h), and S4 (10 h).

**Figure 2:**
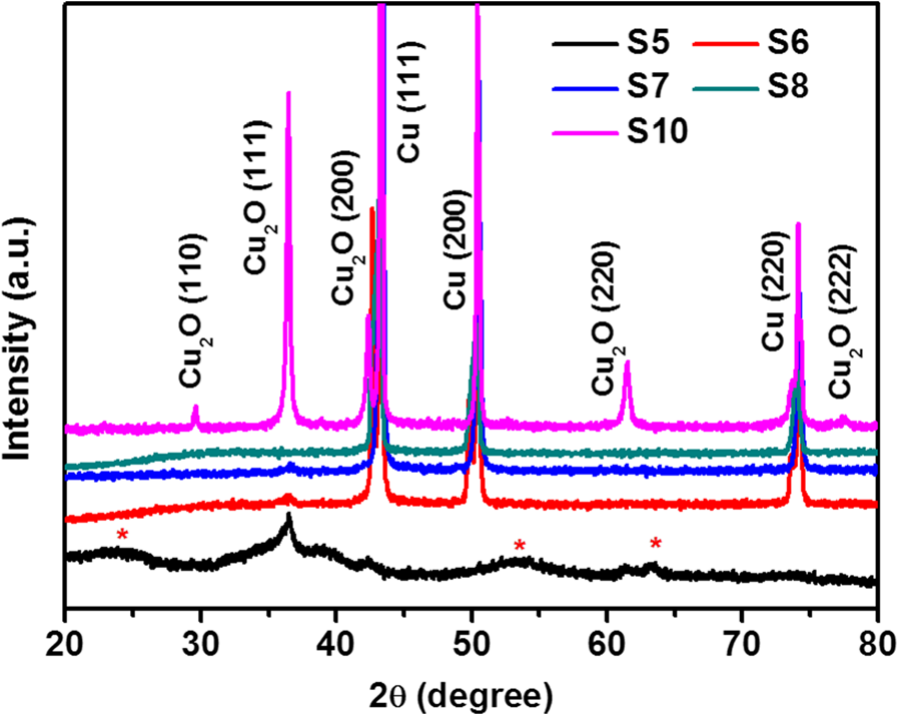
**XRD patterns of as-prepared products obtained at different temperature and copper source.** S5 (4 mmol Cu(CH_3_COO)_2_•H_2_O and 140°C), S6 (4 mmol Cu(CH_3_COO)_2_•H_2_O and 160°C), S7 (4 mmol Cu(CH_3_COO)_2_•H_2_O and 180°C), S8 (4 mmol Cu(NO_3_)_2_⋅3H_2_O and 180°C), and S10 (8 mmol Cu(CH_3_COO)_2_•H_2_O and 160°C). Note that a red star (*) in the figure stands for impurity, which can be indexed to orthorhombic Cu(OH)_2_ according to JCPDS card (no. 80-0656).

Figure 1 shows the XRD patterns of as-prepared products (S1 to S4) obtained at different reaction time. All the peaks of XRD patterns from S2 to S4 can be indexed by ICCD-JCPDS database (card no. 78-2076), which demonstrate that the as-prepared products are the pure Cu_2_O with a cubic symmetry. For the sample S1, the XRD pattern can also be indexed to the cubic Cu_2_O; however, there is a peak around 43.3° being indexed to cubic Cu (111) peak (JCPDS no. 85-1326), which meant that the as-prepared product (S1) is impure Cu_2_O. The average Cu_2_O crystallite size of samples S1 to S4 were calculated to be 11.7 nm (S1), 13.8 nm (S2), 17.4 nm (S3), and 22.3 nm (S4), respectively, by the Debye-Scherer formula combining with Jade 5 software [15,22]. For investigating the effect of reaction time on the phases of obtained products, sample S9 was carried out; however, no precipitant was collected, the final solution kept blue color as the original solution. Thus, reaction time could dominate the phase and control the average crystallite size of the as-prepared samples. Figure 2 depicts the XRD patterns obtained from S5 to S8 and S10 at different temperature and copper sources. Compared the patterns of S5 in Figure 2 and S4 in Figure 1, S4 was pure Cu_2_O while S5 was impure Cu_2_O with poor crystallization, which illustrated that the phase of obtained product was varied when the copper source was changed. With the temperature increasing from 140°C to 180°C (sample S5, S6, and S7), the compositions experienced an evolution from Cu_2_O with impurity (S5), Cu_2_O mixing with Cu (S6), and pure Cu (S7). Pure Cu was also obtained at 180°C (S8) when 4 mmol Cu(NO_3_)_2_⋅3H_2_O was used as copper source. Therefore, temperature played an important role in the composition of as-synthesized products. The influence of copper source amount was also studied by comparing S6 with S10. The composition was not changed (Cu_2_O + Cu), but the ratio of Cu_2_O/Cu increased. In a word, the synthesis conditions, including copper source, temperature, and reaction time, have significant influence on the phases of as-prepared products.

**Figure 3:**
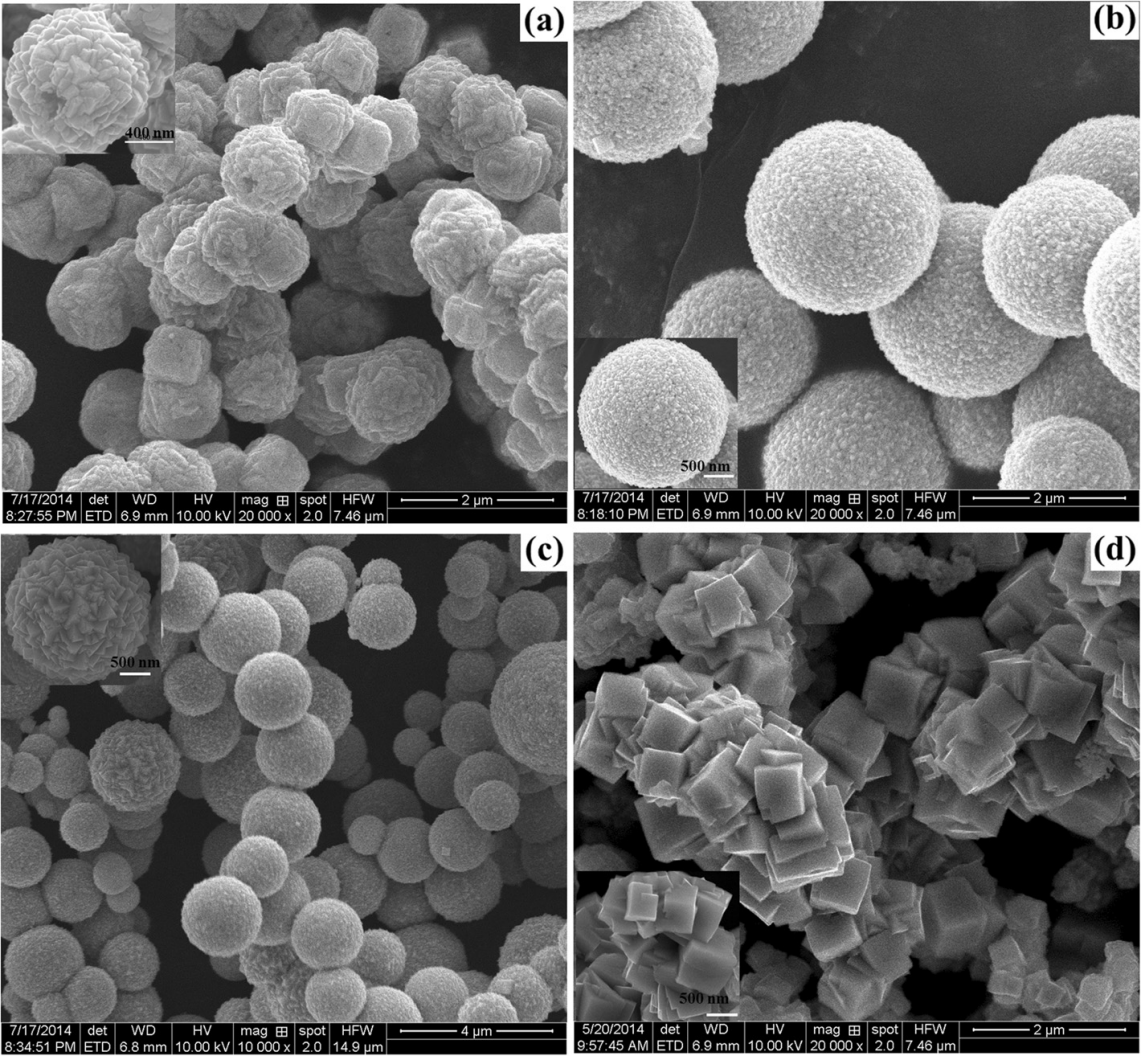
**SEM images of the as-obtained products fabricated at 140°C with different reaction time. (a)** 4 h (S1), **(b)** 6 h (S2), **(c)** 8 h (S3), and **(d)** 10 h (S4) (insets are the corresponding magnified SEM images).

The morphologies of as-prepared samples S1 to S4 under different reaction time are observed by SEM, as shown in Figure 3. The spherical-like crystals were observed with size of 0.5 to 1 μm in Figure 3a for sample S1, which was synthesized at reaction time of 4 h. As increasing reaction time to 6 h, the spherical crystals with rough surface were obtained, as shown in Figure 3b, with the size dispersion of 0.6 to 2.5 μm, which was larger than that of 4 h reaction time. From the inset magnified SEM image, it was easy to find that the sphere was composed of large amount of pyramid particles with diameter of 50 to 100 nm. Figure 3c displays similar morphology as Figure 3b, with the larger spheres of 0.8 to 3.5 μm composing of tremendous pyramids with size of 250 to 300 nm, when the reaction time was further extended to 8 h. As the reaction time reached 10 h, the morphology was completely changed to hierarchical structure consisted of cubic particles with size of 300 to 600 nm, as shown in Figure 3d. The SEM results confirmed that the reaction time had strong influence on the morphological and structural characters of as-obtained products, which were consistent with the calculation from XRD data.

Based on the aforementioned results, we proposed the growth mechanism as follows. For the use of Cu(NO_3_)_2_ · 3H_2_O as copper source, the possible chemical reactions occurred in the system as follows [33,34]:

1 $$ {\mathrm{OHCH}}_2{\mathrm{CH}}_2\mathrm{O}\mathrm{H}\to {\mathrm{CH}}_3\mathrm{C}\mathrm{O}\mathrm{H}+{\mathrm{H}}_2\mathrm{O} $$

2 $$ \mathrm{O}\mathrm{H}-{\mathrm{C}\mathrm{H}}_2-{\mathrm{C}\mathrm{H}}_2-\mathrm{O}\mathrm{H}+{\mathrm{C}\mathrm{u}}^{2+}\to {\left[\mathrm{C}\mathrm{u}\left({\mathrm{C}}_2{\mathrm{H}}_6\mathrm{O}\right)\right]}^{2+} $$

3 $$ {\left[\mathrm{C}\mathrm{u}\left({\mathrm{C}}_2{\mathrm{H}}_6\mathrm{O}\right)\right]}^{2+}+{\mathrm{H}}_2\mathrm{O}\to \mathrm{C}\mathrm{u}{\left(\mathrm{O}\mathrm{H}\right)}_2+{\mathrm{H}\mathrm{OCH}}_2{\mathrm{C}\mathrm{H}}_2\mathrm{O}\mathrm{H}+2{\mathrm{H}}^{+} $$

4 $$ 2\mathrm{C}\mathrm{u}{\left(\mathrm{O}\mathrm{H}\right)}_2+2{\mathrm{CH}}_3\mathrm{C}\mathrm{O}\mathrm{H}\to {\mathrm{Cu}}_2\mathrm{O}+{\mathrm{CH}}_3-\mathrm{C}\mathrm{O}-\mathrm{C}\mathrm{O}-{\mathrm{CH}}_3+3{\mathrm{H}}_2\mathrm{O} $$

5 $$ \mathrm{C}\mathrm{u}{\left(\mathrm{O}\mathrm{H}\right)}_2+{\mathrm{CH}}_3\mathrm{C}\mathrm{O}\mathrm{H}\to \mathrm{C}\mathrm{u}+{\mathrm{CH}}_3\mathrm{CO}\mathrm{O}\mathrm{H}+{\mathrm{H}}_2\mathrm{O} $$

6 $$ 2\mathrm{C}\mathrm{u}+4{\mathrm{CH}}_3\mathrm{CO}\mathrm{O}\mathrm{H}+{\mathrm{O}}_2\to 2\mathrm{C}\mathrm{u}{\left({\mathrm{CH}}_3\mathrm{C}\mathrm{O}\mathrm{O}\right)}_2+2{\mathrm{H}}_2\mathrm{O} $$

Therefore, the reaction listed in Equation 5 would occur for the short time under the suitable temperature, while Equation 6 would happen as more time were needed to dissolve Cu in this system. Herein, Cu phase was contained in the product. For the reactions with longer time (such as S2, S3, and S4), the reactions (Equation 1, 2, 3, 4, 5, and 6) sufficiently occurred and only Cu_2_O phase existed in the products because enough time was supplied to dissolve Cu like Equation 6. However, the higher reaction temperature up to 180°C induced the reaction final stop at the stage of Equation 5; therefore, the final products were pure Cu phase. When Cu(CH_3_COO)_2_ · H_2_O is used as copper source, the aforementioned reactions will occur, but the Cu phase will always appear under higher temperature due to the slow decomposition speed.

As mentioned in previous reports [33,34], spheres are easier to be formed according to the coordinate adsorption, oriented attachment and Ostwald ripening route under solvothermal conditions. In brief, Cu^2+^ ions can be easily formed to a relatively stable complex [Cu(II)EG]^2+^ by chelating reagent ethylene glycol (EG), followed by the slow transformation into Cu(OH)_2_ precursors due to the different stability constants and the sharp decrease of free Cu^2+^ ion concentration, resulting in the separation of nucleation and growth steps. Then, Cu(OH)_2_ could be reduced to Cu_2_O by the acetaldehyde molecules generated from the dehydration of EG. The freshly unstable Cu_2_O pyramids tend to assemble oriented attachments into large spheres driven by the minimization of interfacial energy. As the reaction time increases, the small Cu_2_O pyramids grow larger and change into cubes, and the spheres are broken to form hierarchical structures. Therefore, the temperature plays an important role in the control of compositions of products as well as copper source affects the compositions of products a little bit, while the reaction time has an effective influence on the morphology evolution of products.

**Figure 4:**
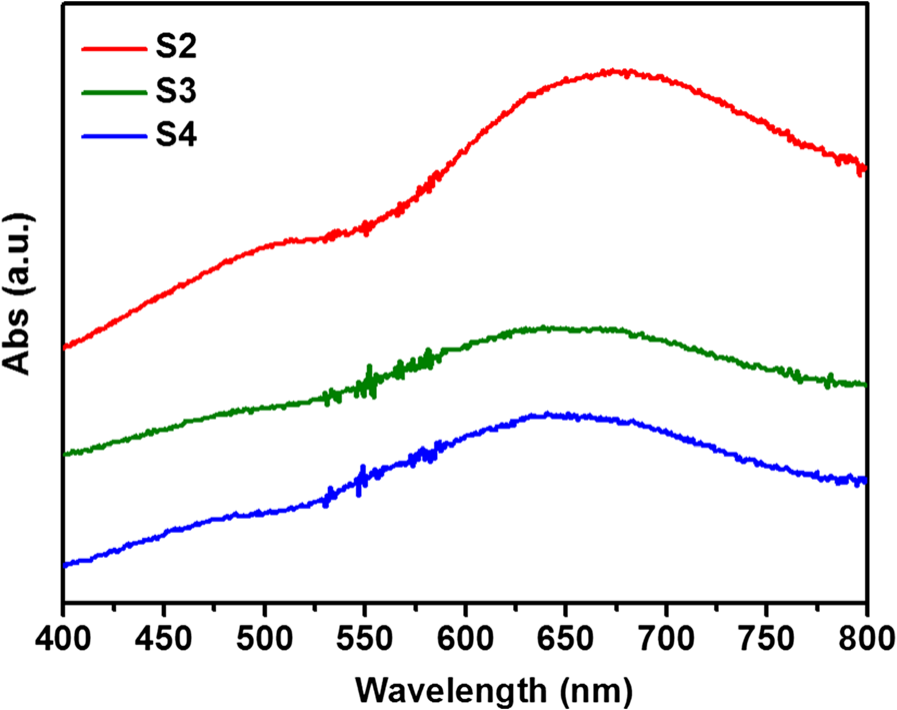
**UV-vis spectra of as-obtained Cu**_**2**_ **O products with different reaction time.** S2 (6 h), S3 (8 h) and S4 (10 h).

The optical absorption behaviors were investigated by UV-vis absorbance spectra, as shown in Figure 4. The optical bandgaps could be affected by the synthetic conditions which are in good agreement with Zhang’s report [35]. The strong light scattering bands resulted from the sizes of the samples, which were observed dominantly in the absorption spectra for the samples S2, S3 and S4, similar to the previous report [26]. These light scattering bands and absorption bands show progressive blue shifts with the increase of the size for S2, S3, and S4. The bandgap energy (E_g_) calculated from the absorption at 450 to 525 nm is in the range of 2.36 to 2.76 eV, which is consistent with other reports [17,22,26]. The values greater than that of bulk Cu_2_O at 2.17 eV are attributed to the quantum confinement effects [22,26]. These results confirmed that the as-prepared Cu_2_O products were suitable candidates for photocatalysts under visible range of the solar spectrum.

**Figure 5:**
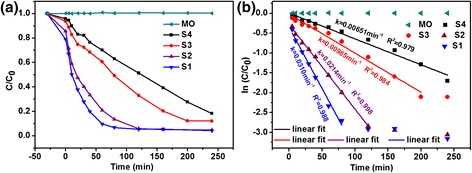
**Photocatalytic activity of as-obtained Cu**_**2**_ **O products under visible light. (a)** Plots of concentration ratios of MO in an aqueous solution against given irradiation intervals in the presence or absence of Cu_2_O products for samples S1 to S4, which depicted the synthesis conditions as listed in Table 1. **(b)** The plots of ln(*C*/*C*_0_) versus time of MO degradation in presence of Cu_2_O products, without H_2_O_2_.

Figure 5 shows the photocatalytic activities of as-obtained Cu_2_O products without H_2_O_2_ additions (UV-vis spectral variations of MO in an aqueous solution were shown in Additional file 1: Figure SI-1). It is found from Figure 5A that the concentrations of MO decrease continually with an increase of irradiation duration under visible light for samples S1 to S4 with Cu_2_O products while MO is kept almost no change for the pure MO solution. The different ratios of MO degradation are also observed which can be attributed to the morphology difference. The pseudo-first-order kinetics model was used to determine the rate constant of photodegradation of MO with respect to the degradation time [3,6]:

$$ ln\kern0.24em \left(C/{C}_0\right)=-kt $$

where *C*0 is the initial concentration of MO and *C* is the concentration at time *t*, *k* is the reaction rate constant. The plots of ln(*C/C*0) versus time *t* for MO degradation using Cu_2_O products were illustrated in Figure 5B. The rate constants were given by the slopes of linear lines and estimated to be 0.031 min^−1^, 0.0214 min^−1^, 0.00985 min^−1^, and 0.00651 min^−1^ for samples S1, S2, S3, and S4, respectively. The obtained values demonstrated that the degradation rates for MO followed the order of S1 > S2 > S3 > S4 without the addition of H_2_O_2_. The degradation rate can reach 93% without the addition of H_2_O_2_, which is higher than other reports such as Cu_2_O nanoparticles [3], Cu_2_O microcrystals [27], Cu_2_O-graphene [36], and pure CuO microsphere and CuO/Cu_2_O microspheres [37].

**Figure 6:**
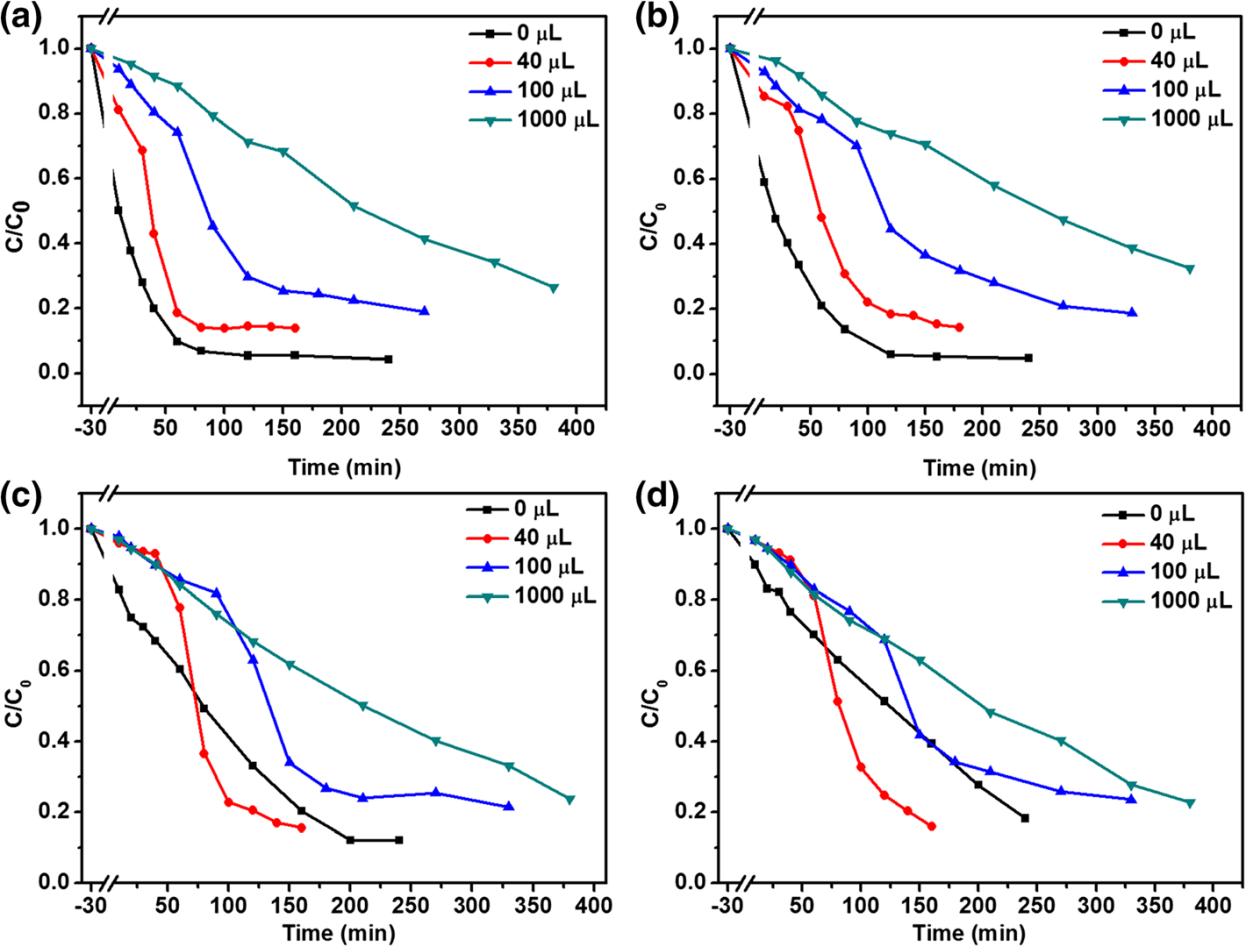
**Photodegradation of MO in an aqueous solution with Cu**_**2**_ **O products and different amount of H**_**2**_ **O**_**2**_ **. (a)** 4 h (S1), **(b)** 6 h (S2), **(c)** 8 h (S3), and **(d)** 10 h (S4).

**Figure 7:**
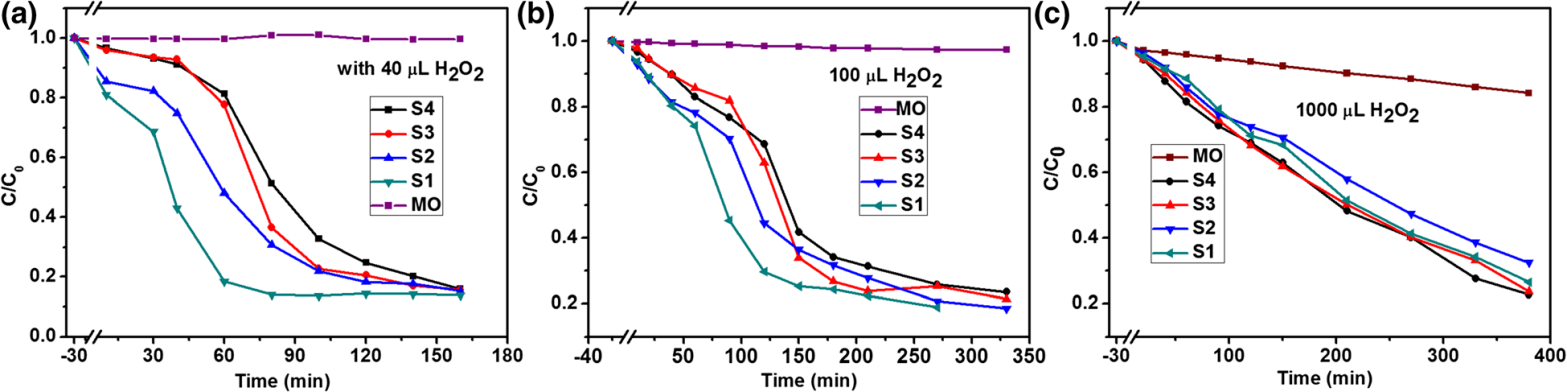
**Photocatalytic activity of as-obtained Cu**_**2**_ **O products with different amount of H**_**2**_ **O**_**2**_ **.** Plots of concentration ratio of MO in an aqueous solution against given irradiation intervals in the presence of Cu_2_O products with different amount of H_2_O_2_: **(a)** 40 μL, **(b)** 100 μL, and **(c)** 1,000 μL.

The effects of H_2_O_2_ amount on the photocatalytic activities are presented in Figure 6. The UV-vis absorption spectra of MO in an aqueous solution with different amount of H_2_O_2_ and in the presence of Cu_2_O products with different reaction time were shown in Additional file 1: FigureSI-2, SI-3, and SI-4. The degradation rate of 86% was observed with 40 μL H_2_O_2_ which is higher than other reports such as Cu_2_O nanoparticles [3], Cu_2_O/CuO hollow microspheres [38], micro-nanohierarchical Cu_2_O structure [21], and Cu_2_O microcrystalline particle film [24]. For samples S1 (Figure 6A) and S2 (Figure 6B), the photocatalytic activities were inhibited when the amount of H_2_O_2_ were increased from 0 μL to 1,000 μL. However, for samples S3 (Figure 6C) and S4 (Figure 6D), the different behaviors were observed compared with S1 and S2. For sample S3 (8 h), the photodegradation rate with the addition of 40 μL H_2_O_2_ achieved maximum, as shown in Figure 6C, which is the same to sample S4. There is no obvious difference with increase of the amounts of H_2_O_2_. On the other hand, the effects of reaction time were also investigated on the photodegradation of MO in an aqueous solution with different amounts of H_2_O_2_, as shown in Figure 7. The corresponding curves of ln(*C/C*0) versus time *t* for MO degradation using Cu_2_O products were plotted in Additional file 1: FigureSI-5. Combining with Figure 5A, the gap of the effect of reaction time on the photodegradation rate was rapidly closing when the amount of H_2_O_2_ was increased, which meant that the morphology effect on the photocatalytic activity became weaker by increasing the addition of H_2_O_2_ amount. The results are slightly different from the previous reports [23,24], which can be attributed to the competition between the effect of H_2_O_2_ and the morphology of Cu_2_O photocatalysts. That means H_2_O_2_ plays a dominant role in the process of photodegradation when a mass of H_2_O_2_ and lager-sized photocatalysts are used.

The mechanism of a possible photochemical reaction was proposed according to the factors influenced on the photocatalytical activity. The electron can excite from valence band (VB) to conductance band (CB) under visible light irradiation to the surface of Cu_2_O, as shown in Equation 1, and thus, a series of reactions could be induced by the photogenerated electrons and holes as follows [23,24,29,39]:

7 $$ {\mathrm{Cu}}_2\mathrm{O}+hv\to {e}^{-}\left({\mathrm{CBCu}}_2\mathrm{O}\right)+{h}^{+}\left({\mathrm{VBCu}}_2\mathrm{O}\right) $$

8 $$ {\mathrm{O}}_2+{e}^{-}\to \cdot {\mathrm{O}}_2^{-} $$

9 $$ {\mathrm{O}}_2+2{\mathrm{H}}_2\mathrm{O}+2{e}^{-}\to {\mathrm{H}}_2{\mathrm{O}}_2+2{\mathrm{O}\mathrm{H}}^{-} $$

10 $$ {\mathrm{H}}_2{\mathrm{O}}_2+\cdot {\mathrm{O}}_2^{-}\to \cdot \mathrm{O}\mathrm{H}+{\mathrm{O}\mathrm{H}}^{-}+{\mathrm{O}}_2 $$

11 $$ {h}^{+}+{\mathrm{H}}_2\mathrm{O}\to \cdot \mathrm{O}\mathrm{H}+{\mathrm{H}}^{+} $$

12 $$ \cdot \mathrm{O}\mathrm{H}+\mathrm{M}\mathrm{O}\to \mathrm{degradation}\kern0.24em \mathrm{products} $$

For the degradation of MO in the presence of Cu_2_O without the addition of H_2_O_2_ as shown in Figure 5A, the photocatalytic reaction can be as follows based on the previous report [21,23]: First, the photogenerated electrons and holes were formed under visible irradiation of Cu_2_O, as depicted in Equation 7; Secondly, the electrons were scavenged by oxygen (O_2_) to generate superoxide ions $\left({O}_2^{-}\right)$ (Equation 8) and H_2_O_2_ (Equation 9). Then, ${O}_2^{-}$ reacted with H_2_O_2_ to produce hydroxyl radicals (•OH) (Equation 10). The holes reacted also with H_2_O to produce hydroxyl radicals (•OH), as displayed in Equation 11. Finally, the pollutants (MO) were oxidized into inorganic or nontoxic products by hydroxyl radicals (•OH) (Equation 12). The difference of photodegradation rate of samples S1 to S4 could be ascribed to the morphological character [39,40]. It was well known that the surface area and surface state strongly affected the activity of photocatalyst due to the photocatalytic reaction always taking place at the surface of photocatalyst [39,40]. The specific surface area of as-obtained Cu_2_O products were evaluated to be 8.44 m^2^/g, 5.50 m^2^/g, 4.68 m^2^/g, and 5.07 m^2^/g for samples S1, S2, S3, and S4 (as shown in Additional file 1: Figure SI-7 and Table SI-1). Although the total surface area of sample S4 was higher than that of S3, the photocatalytic activity of S4 lower, which may be ascribed to more [111] surface on sample S3 [29]. Another reason may be ascribed to the difference of bandgap, the bandgap energies (E_g_) calculated from the absorption at 450 to 525 nm are in the range of 2.36 to 2.76 eV for samples S2, S3, and S4, which means that more photogenerated electrons and holes occur on the surfaces of samples with the order of S2 > S3 > S4 for the same irradiation condition. Moreover, sample S1 contains a small mass of Cu, which can be identified from XRD pattern. This Cu phase may also enhance the photodegradation by promoting the rapid separation of photogenerated electrons and holes in the interfaces between Cu and Cu_2_O [41,42].

Once adding H_2_O_2_ into the aqueous solution, the photochemical process became complicated. H_2_O_2_ was considered to be a good electron acceptor by numerous studies, and thus it could be converted to •OH by accepting electrons (Equation 10) [23]. Based on this view, the addition of H_2_O_2_ with a suitable amount should enhance the photodegradation rate which was confirmed in our experiments, as shown in Figure 6C,D, while the results in Figure 6A,B were inconsistent with this view. From Additional file 1: FigureSI-6, it can be seen that the photodegradation rate was continually enhanced by increasing of H_2_O_2_ in the absence of Cu_2_O products for decomposition of MO in an aqueous solution, although the absolute values of photodegradation rates were very small. Therefore, both H_2_O_2_ and Cu_2_O could enhance the photodegradation of MO in an aqueous solution as aforementioned, respectively. However, when the two products were mixed together and placed into MO solution, the photocatalytic activity was not further enhanced in some cases. This may result from the photochemical reaction between H_2_O_2_ and Cu_2_O, as follows [23,43]:

13 $$ 2{\mathrm{H}}_2{\mathrm{O}}_2\overset{{\mathrm{Cu}}_2\mathrm{O}}{\to }2{\mathrm{H}}_2\mathrm{O}+{\mathrm{O}}_2 $$

14 $$ {\mathrm{Cu}}_2\mathrm{O}+{\mathrm{H}}_2{\mathrm{O}}_2\to 2\mathrm{C}\mathrm{u}\mathrm{O}+{\mathrm{H}}_2\mathrm{O}, $$

which indicated that H_2_O_2_ would be decomposed into O_2_ by Cu_2_O (Equation 13) and would react with Cu_2_O to produce CuO (Equation 14) depending on the amount of H_2_O_2_ and the morphology of Cu_2_O product. According to the previous report [44], the photocorrosion of Cu_2_O will induce them changing into CuO, resulting in the loss of photogenerated charges, and herein, the photodegradation rate decreases. The reaction (Equation 14) preferably occurs at the surfaces of nanosized particles due to its high activity, which means the amount of H_2_O_2_ would affect the samples’ photodegradation rate with the sequence of S1 > S2 > S3 > S4. Therefore, the final photocatalytic activity on the photodegradation of MO in an aqueous solution should be determined by both H_2_O_2_ and Cu_2_O. As for the effects of H_2_O_2_, they act as inhibitor of the photodegradation for the samples with smaller size but work as activator while the photocatalysts with large size are used. Therefore, both the additive H_2_O_2_ and morphology of Cu_2_O products played important roles in the photocatalytic activity, the final efficiency could be determined by the competition of the effect of H_2_O_2_ and morphology.

## Conclusions

In summary, hierarchically nanostructured Cu_2_O samples were successfully synthesized by a solvothermal method. The structural and morphological characters were investigated by XRD and SEM to prove that the synthesis conditions had significant influence on the composition of products and the formation of crystal with diverse morphologies. The specific surface areas of as-obtained samples were also observed to explain the difference of photodegradation rate with as-obtained samples. The amount of H_2_O_2_ additive was confirmed to play an important role in the photodegradation of MO as well as morphology under visible light irradiation. It was revealed that the photocatalytic activities were of comprehensive effect of the amount of H_2_O_2_ and the morphology of Cu_2_O photocatalysts.

## Additional file

Additional file 1:
**Supporting information.** FigureSI-1, FigureSI-2, FigureSI-3, FigureSI-4, FigureSI-5, FigureSI-6, FigureSI-7, and TableSI-1.
