# Supplementary material for: Effects of architectures and H2O2 additions on the photocatalytic performance of hierarchical Cu2O nanostructures
Source: Nanoscale Res Lett. 2015 Jan 22;10:8. doi: 10.1186/s11671-014-0726-x (PMC4311901; doi:10.1186/s11671-014-0726-x)
Supplement: Additional file 1: — Supporting information. FigureSI-1, FigureSI-2, FigureSI-3, FigureSI-4, FigureSI-5, FigureSI-6, FigureSI-7, and TableSI-1. [file 11671_2014_726_MOESM1_ESM.doc]

Supporting information

**Effects of architectures and H_2_O_2_ additions on the photocatalytic performance of hierarchical Cu_2_O nanostructures**

Xiaolong Deng, ^†^* Qiang Zhang, ^†^Qinqin Zhao, ^†^Lisha Ma, ^†^Meng Ding^†^ and Xijin Xu^†^*

^†^School of Physics and Technology, University of Jinan, 336 NanxinZhuang West Road, Jinan, 250022, Shandong Province, Peoples Republic of China

*To whom correspondence should be addressed:sps_dengxl@ujn.edu.cn and sps_xuxj@ujn.edu.cn.

1. **Time-dependent UV-vis absorption spectra with the change of an aqueous solution of MO in the presence of Cu_2_O products, without H_2_O_2_, with different reaction time of synthesis condition**


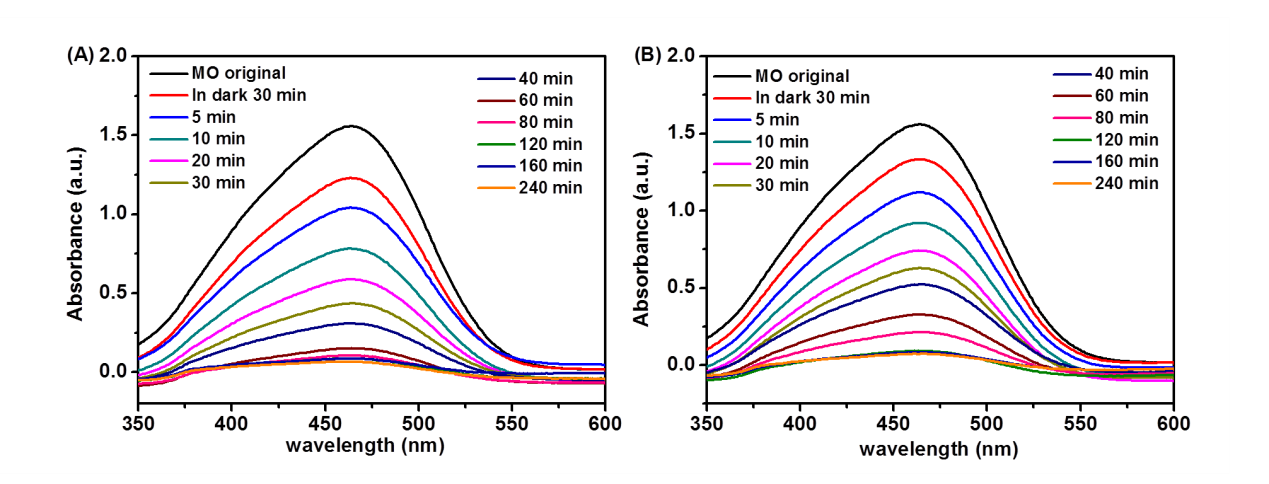

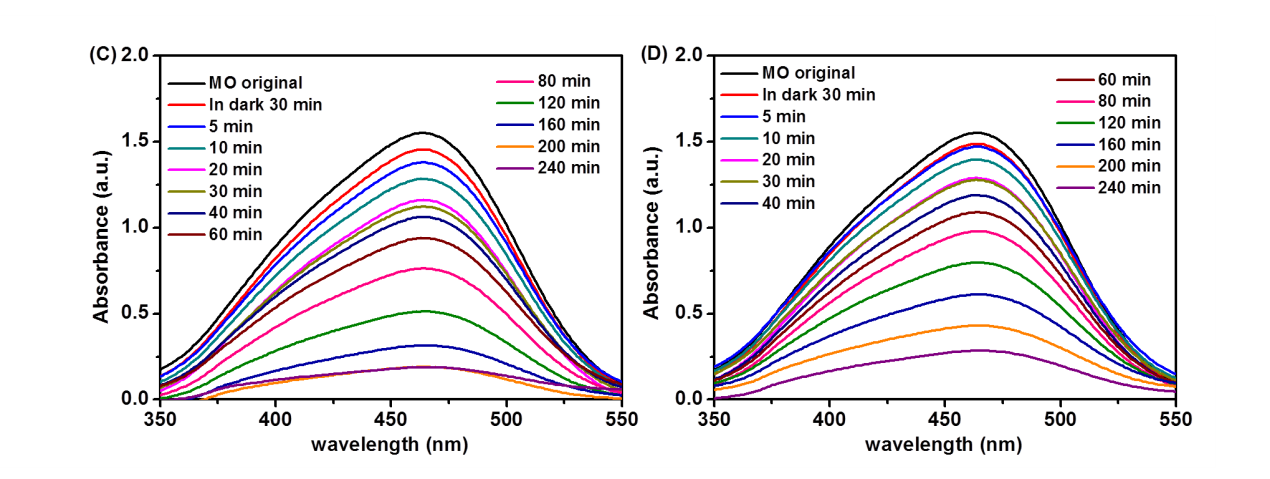


**FigureSI-1Time-dependent UV-vis absorption spectra with the change of an aqueous solution of MO**.Time-dependent UV-vis absorption spectra with the change of an aqueous solution of MO in the presence of Cu_2_O products, without H_2_O_2_, with different reaction time of synthesis condition: (A) 4 h (S1), (B) 6 h (S2), (C) 8 h (S3), and (D) 10 h (S4).

1. **Time-dependent UV-vis absorption spectra with the change of an aqueous solution of MO in the presence of Cu_2_O products with H_2_O_2_ and different reaction time**


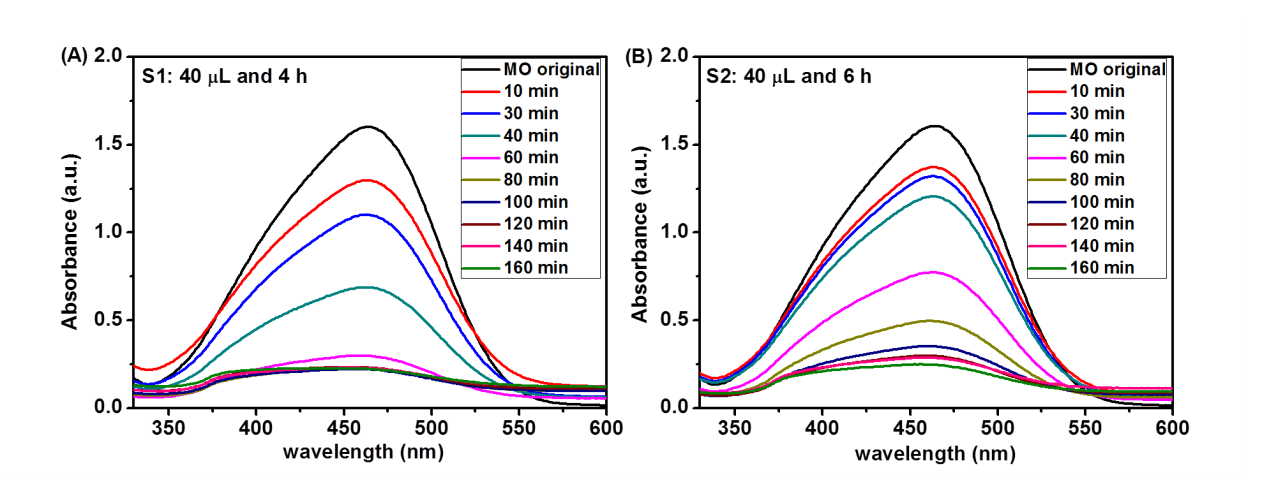

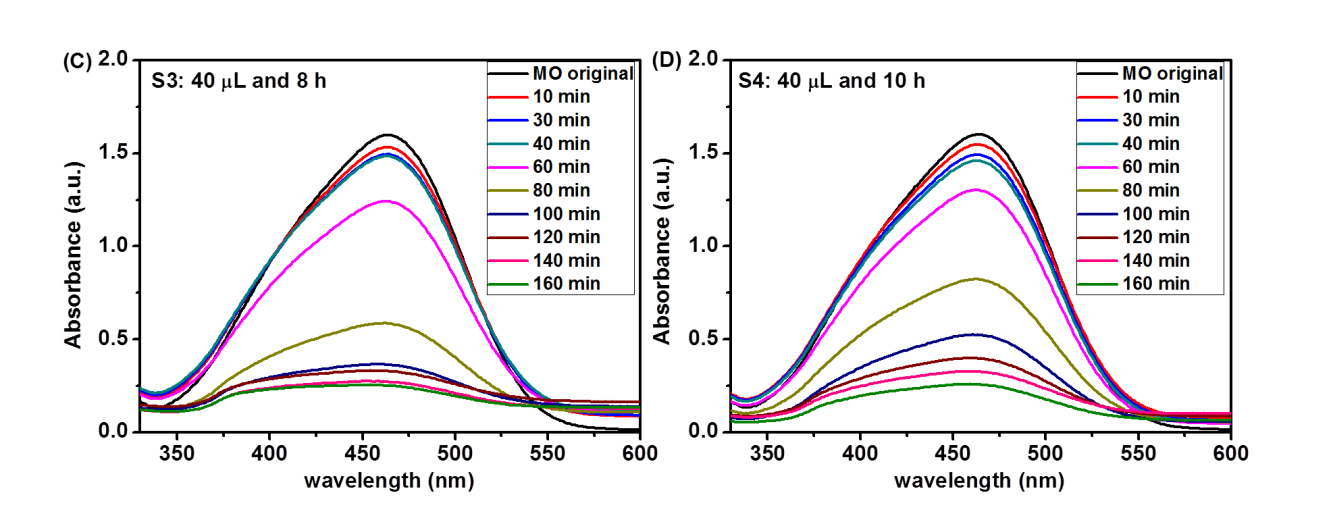


**FigureSI-2Time-dependent UV-vis absorption spectra with the change of an aqueous solution of MO**. Time-dependent UV-vis absorption spectra with the change of an aqueous solution of MO in the presence of Cu_2_O products with H_2_O_2_ and different reaction time: (A) 40 µL and 4 h (S1), (B) 40 µL and 6 h (S2), (C) 40 µL and 8 h (S3), and (D) 40 µL and 10 h (S4).

1. **Time-dependent UV-vis absorption spectra with the change of an aqueous solution of MO in the presence of Cu_2_O products with H_2_O_2_ and different reaction time**


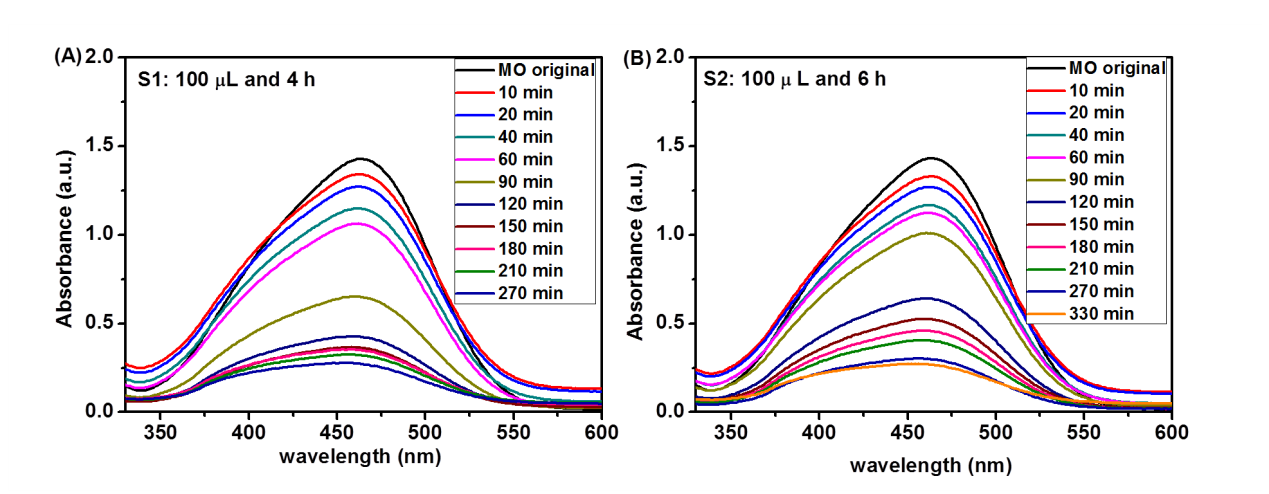

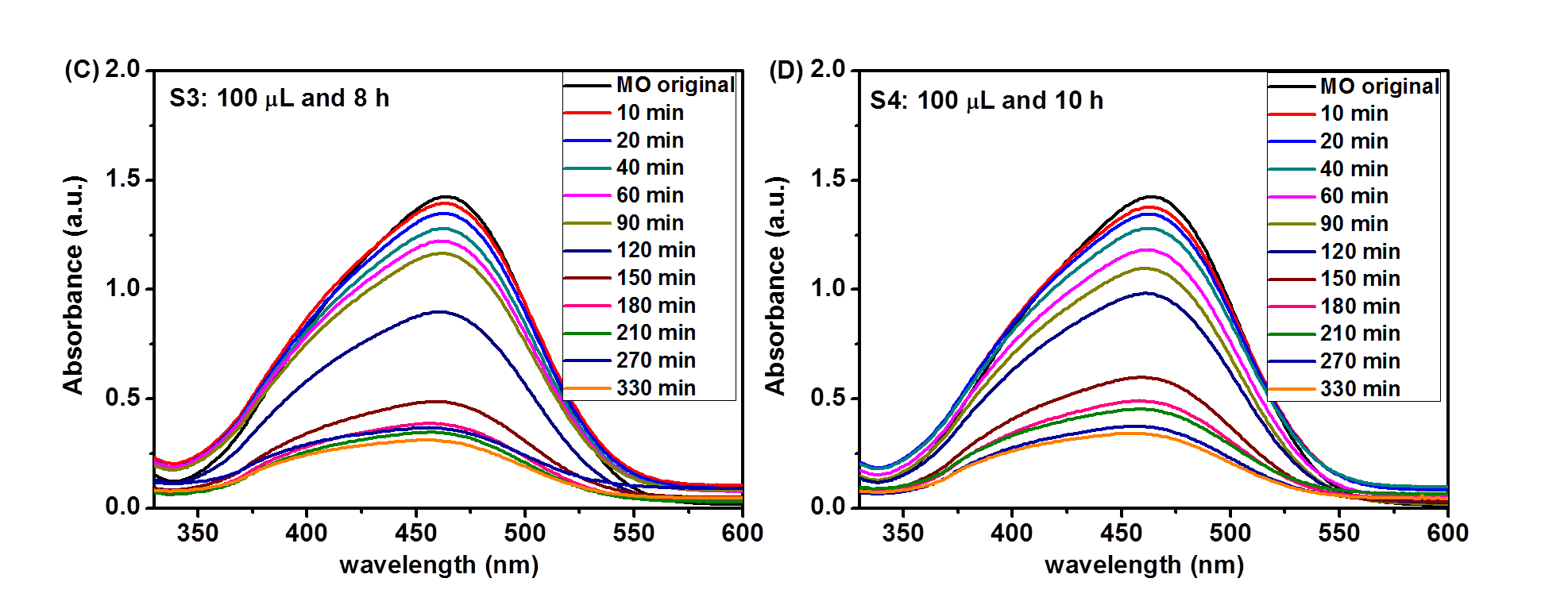


**FigureSI-3Time-dependent UV-vis absorption spectra with the change of an aqueous solution of MO**.Time-dependent UV-vis absorption spectra with the change of an aqueous solution of MO in the presence of Cu_2_O products with H_2_O_2_ and different reaction time: (A) 100 µL and 4 h (S1), (B) 100 µL and 6 h (S2), (C) 100 µL and 8 h (S3), and (D) 100 µL and 10 h (S4).

1. **Time-dependent UV-vis absorption spectra with the change of an aqueous solution of MO in the presence of Cu_2_O products with H_2_O_2_ and different reaction time**


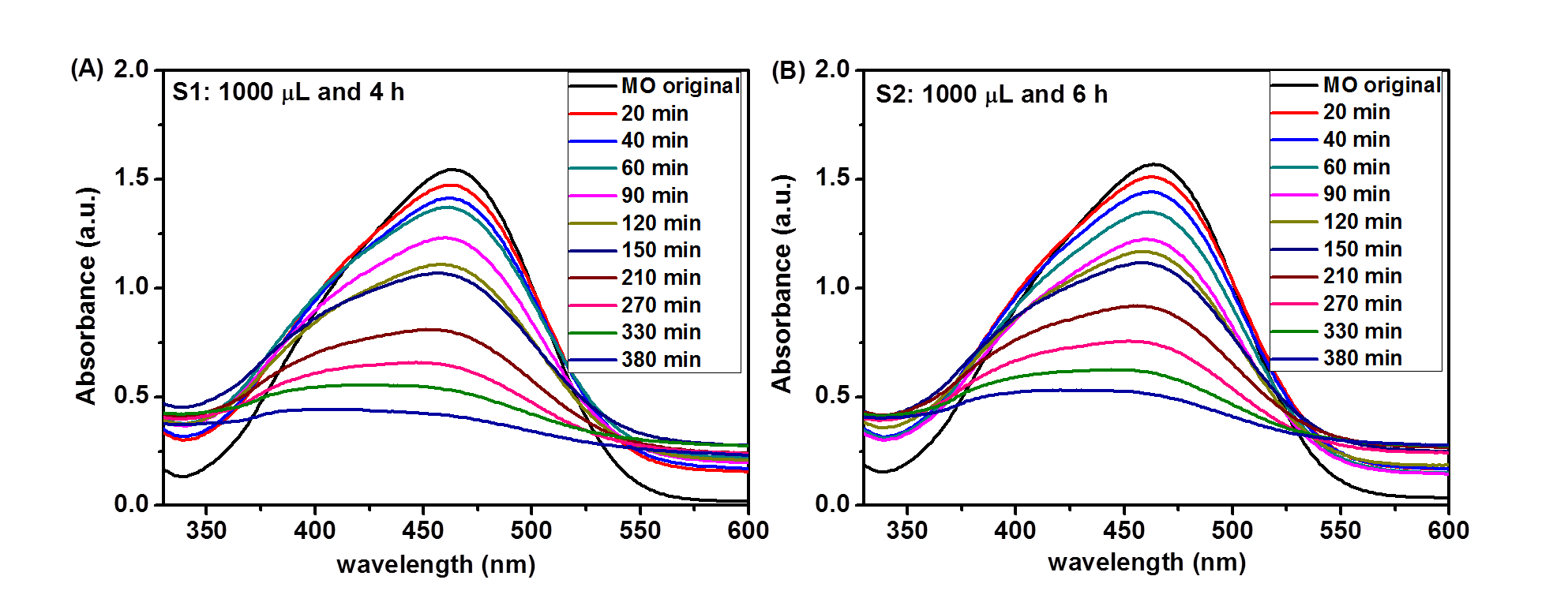

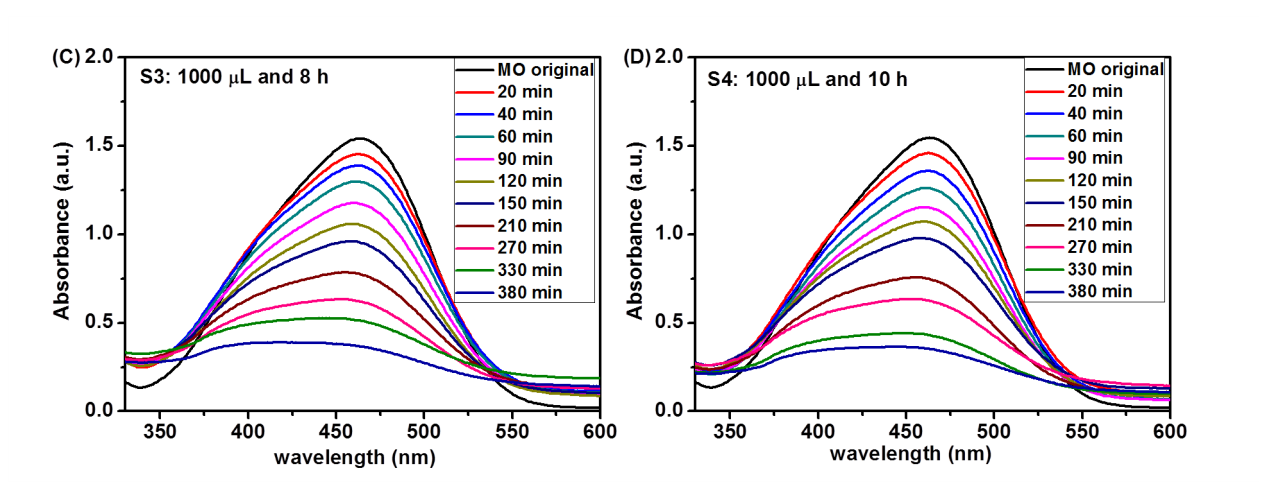


**FigureSI-4Time-dependent UV-vis absorption spectra with the change of an aqueous solution of MO**.Time-dependent UV-vis absorption spectra with the change of an aqueous solution of MO in the presence of Cu_2_O products with H_2_O_2_ and different reaction time: (A) 1000 µL and 4 h (S1), (B) 1000 µL and 6 h (S2), (C) 1000 µL and 8 h (S3), and (D) 1000 µL and 10 h (S4).

1. **Plots of ln(C/C_0_) versus time of MO degradation with different amount of H_2_O_2_ and in presence of Cu_2_O products with various reaction time**


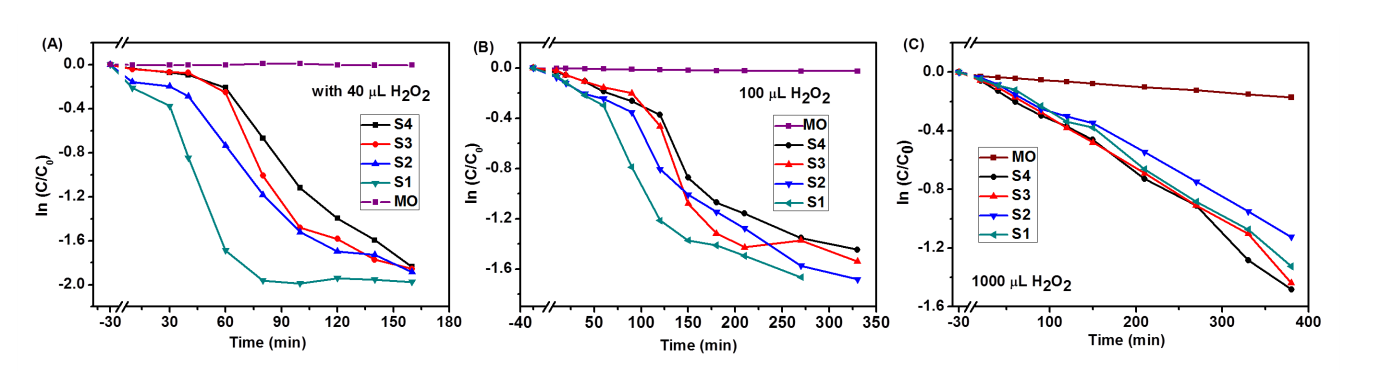


**FigureSI-5Photocatalytic activity of as-obtained Cu_2_O products with different amount of H_2_O_2_.**Plots of ln(C/C_0_) versus time of MO degradation with different amount of H_2_O_2_ and in presence of Cu_2_O products with various reaction time: (A) 40 µL, (B) 100 µL, and (C) 1000 µL.

1. **Plots of concentration ratio and ln(C/C_0_) versus time of MO in an aqueous solution against given irradiation intervals in the presence of H_2_O_2_ and in the absence of Cu_2_O products.**

The variation of concentration ratio of MO in an aqueous solution against given irradiation intervals were plotted,as shownin FigureSI-6A, for control sample with different amount ofH_2_O_2_in the absence of Cu_2_O product. It was revealed that the photocatalytic activity was increased with the increase of H_2_O_2_ amount. The photodegradation rates were plotted in FigureSI-6B, which showed that the same tendency and confirmed the results as depicted in FigureSI-6A, although the values were very small.


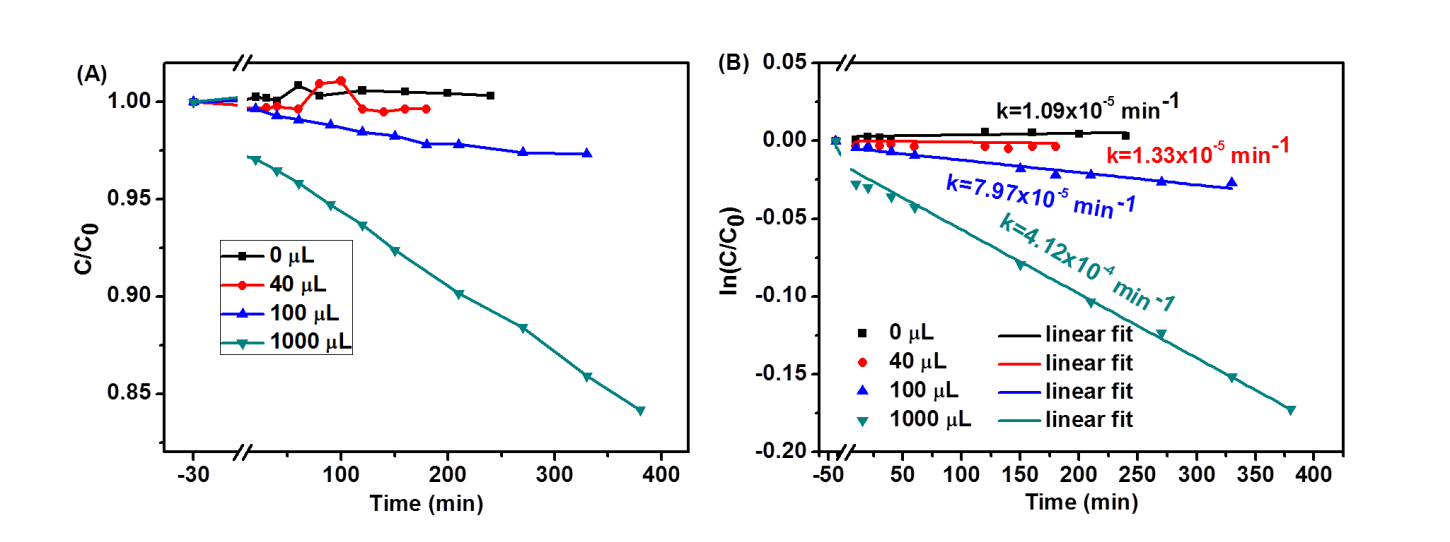


**FigureSI-6Photodegradation of MO under visible light irradiation with different conditions.** (A) Plots of concentration ratio of MO in an aqueous solution against given irradiation intervals in the presence of H_2_O_2_ and in the absence of Cu_2_O products. (B) The plots of ln(*C/C_0_*) versus time of MO degradation in the presence H_2_O_2_ without Cu_2_O products.

1. **Specific surface area of the products measurement results.**

The Brunauer–Emmett–Teller (BET) specific surface areas of the products were investigated by N_2_ adsorption isotherm using a full-automatic specific surface analyzer (3H-2000BET-A, Beishide Instrument, Beijing, China).The surface areas of the products were evaluated by comparing with a standard reference sample, as shown in Figure SI-7 and Table SI-1.


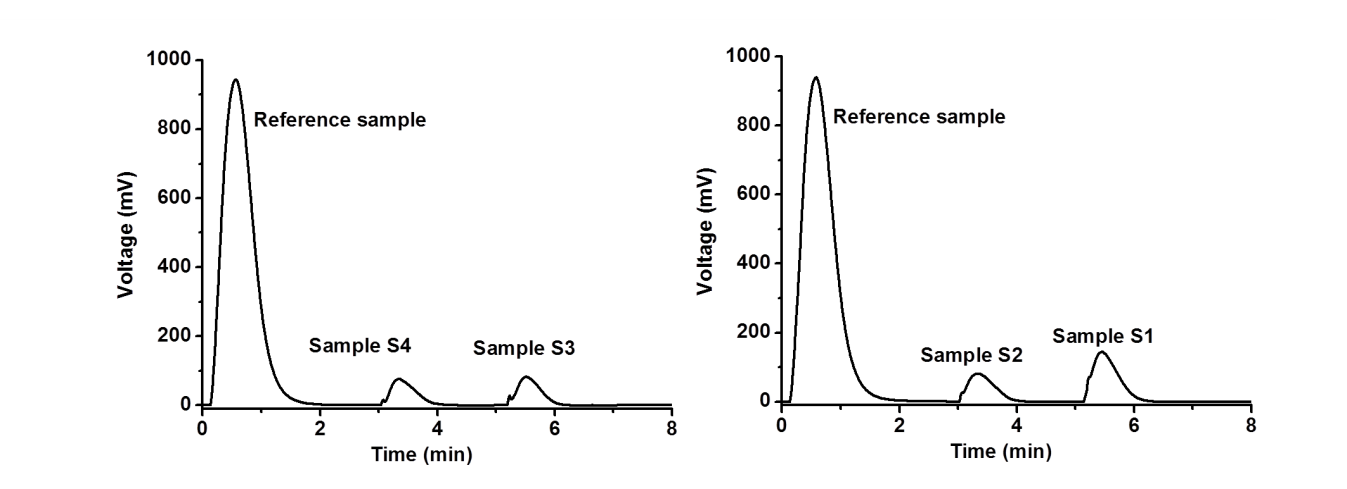


**Figure SI-7 Specific surface area measurement of as-prepared products by N_2_ adsorption compared with the standard reference sample.**

**Table SI-1 Specific surface area results of as-prepared products**

| Sample name | Weight (mg) | Peak area | Specific surface area (m^2^/g) |
| --- | --- | --- | --- |
| Reference sample | 2383.7 | 1740926 | 9.6 |
| S1 | 342.6 | 220101 | 8.44 |
| S2 | 309.6 | 129630 | 5.5 |
| S3 | 317.3 | 113030 | 4.68 |
| S4 | 283 | 109182 | 5.07 |
